# Isorhapontigenin inhibition of basal muscle-invasive bladder cancer attributed to its downregulation of SNHG1 and DNMT3b

**DOI:** 10.1186/s12885-024-12490-5

**Published:** 2024-06-15

**Authors:** Hao Meng, Rui Yang, Qianqian Lin, Wenqi Du, Zheng Chu, Yaxin Cao, Mengxiang Du, Yazhen Zhao, Jiheng Xu, Ziyi Yang, Xiaomin Xie, Lijiong He, Chuanshu Huang

**Affiliations:** 1 Key Laboratory of Medicine, Ministry of Education, School of Laboratory Medicine and Life Sciences, Wenzhou Medical University, Wenzhou, Zhejiang 325035 China; 2 Oujiang Laboratory (Zhejiang Lab for Regenerative Medicine, Vision and Brain Health), Wenzhou Zhejiang, 325053 China

**Keywords:** ISO, SNHG1, DNMT3b, miR-129, Basal bladder cancer

## Abstract

**Background:**

Bladder cancer (BC) is among the most prevalent malignant urothelial tumors globally, yet the prognosis for patients with muscle-invasive bladder cancer (MIBC) remains dismal, with a very poor 5-year survival rate. Consequently, identifying more effective and less toxic chemotherapeutic alternatives is critical for enhancing clinical outcomes for BC patients. Isorhapontigenin (ISO), a novel stilbene isolated from a *Gnetum* found in certain provinces of China, has shown potential as an anticancer agent due to its diverse anticancer activities. Despite its promising profile, the specific anticancer effects of ISO on BC and the underlying mechanisms are still largely unexplored.

**Methods:**

The anchorage-independent growth, migration and invasion of BC cells were assessed by soft agar and transwell invasion assays, respectively. The RNA levels of SOX2, miR-129 and SNHG1 were quantified by qRT-PCR, while the protein expression levels were validated through Western blotting. Furthermore, methylation-specific PCR was employed to assess the methylation status of the miR-129 promoter. Functional assays utilized siRNA knockdown, plasmid-mediated overexpression, and chemical inhibition approaches.

**Results:**

Our study demonstrated that ISO treatment significantly reduced SNHG1 expression in a dose- and time-dependent manner in BC cells, leading to the inhibition of anchorage-independent growth and invasion in human basal MIBC cells. This effect was accompanied by the downregulation of MMP-2 and MMP-9 and the upregulation of the tumor suppressor PTEN. Further mechanistic investigations revealed that SOX2, a key upstream regulator of SNHG1, played a crucial role in mediating the ISO-induced transcriptional suppression of SNHG1. Additionally, we found that ISO treatment led to a decrease in DNMT3b protein levels, which in turn mediated the hypomethylation of the miR-129 promoter and the subsequent suppression of SOX2 mRNA 3’-UTR activity, highlighting a novel pathway through which ISO exerts its anticancer effects.

**Conclusions:**

Collectively, our study highlights the critical role of SNHG1 downregulation as well as its upstream DNMT3b/miR-129/SOX2 axis in mediating ISO anticancer activity. These findings not only elucidate the mechanism of action of ISO but also suggest novel targets for BC therapy.

## Introduction

BC is one of the most common malignant urothelial tumors in the world [1, 2]. The most common type of BC is non-muscle invasive bladder cancer (NMIBC), which accounts for 75% of newly diagnosed BC; MIBC accounts for 25% of all new diagnoses [3]. To date, clinical treatment of NMIBC involves transurethral resection of a bladder tumor (TURBT), combined with adjuvant intravesical therapy (IVe) based on a risk-stratified approach, achieving an overall survival rate of 90% [4]**.** However, due to various factors such as tumor heterogeneity, which leads to unique pathological and molecular characteristics, the cure rate for MIBC remains very poor, especially in patients with distant metastases [4]**.** Therefore, identifying new molecules responsible for mediating human BC development and finding more effective and less toxic alternative chemotherapeutic therapies are critical for improving the clinical outcomes of BC patients.

ISO is a novel derivative of stilbene isolated from the Chinese herb *Gnetum* and is a potential anticancer drug used as a therapeutic regimen for BC patients [5–7]. Its chemical structure [6, 7] and multiple anticancer activities [8–13] have been demonstrated in our previous studies. At a low dose (5–10 µM), ISO induced cell cycle arrest at the G0/G1 phase and inhibited anchorage-independent cell growth through downregulation of cyclin D1 and upregulation of p27 [7, 11]. Further study indicated that cyclin D1 inhibition by ISO is mediated by the miR-137/Sp1 pathway in UM-UC-3 and T24T cells [8]. At higher doses (40–60 µM), ISO induced cell apoptosis by downregulating the X-linked inhibitor of apoptosis protein (XIAP) in T24T cells [6]. In addition, our studies showed that ISO specifically suppresses the invasion of UM-UC-3 and T24T cells in vitro by targeting the STAT1/FOXO1/MMP-2 axis [9] and the miR-137/GSK3β/HSP70/MMP-2 axis [12] and inhibits the development of invasive BC in mice following exposure to the bladder carcinogen N-butyl-N-(4-hydroxybutyl) nitrosamine (BBN) in vivo [9]. Moreover, ISO treatment induces autophagy and inhibits cell growth in UM-UC-3 cells through JNKs/JUN-dependent transcriptional induction of SESN2 [10]. Finally, our recent studies showed that ISO inhibits the stem cell-like properties and invasion of BC by attenuating CD44 expression [13]. Taken together, our results suggest that ISO might be a promising agent for the prevention and treatment of human BCs.

Noncoding RNAs (ncRNAs) include ribosomal RNA (rRNA) and other types that can be divided into short and long noncoding RNAs (lncRNAs), including antisense RNA (asRNA), pseudogenes, long intergenic ncRNA (lincRNA), and circular RNA (circRNA) [14]. LncRNAs were once thought to be “junk RNAs”, but an increasing number of studies have reported that they play important molecular and functional roles in various human cancers [15]. Aberrant expression of lncRNAs has been associated with multiple steps of human cancer development, including cell proliferation, migration, invasion, and metastasis [16–18]. Due to their high tissue specificity, sensitivity and reliable stability, lncRNAs are promising tumor biomarkers and therapeutic targets for cancer treatment [19].

Among lncRNAs, small nucleolar RNA host gene 1 (SNHG1) is a novel oncogenic lncRNA aberrantly expressed in various types of human cancers [20–28], including hepatocellular carcinoma [20, 29], esophageal squamous cell carcinoma [21], gastric cancer [23], breast cancer [24], prostate cancer [25], acute myeloid leukemia [26], non-small cell lung cancer [27] and colorectal cancer [28]. Nevertheless, the roles, mechanisms, and upstream regulator/downstream effectors of SNHG1 in human MIBC invasion and growth have rarely been explored. Therefore, there is an urgent need to elucidate the molecular functions and cellular mechanisms of SNHG1 in human MIBC. In the present study, we addressed these critical questions and investigated the potential inhibitory effects of ISO on invasion and growth of human MIBC cells, with a specific focus on the role of SNHG1.

## Materials and methods

### Cell Culture and transfections

The invasive human BC cell lines, 5637 and UM-UC-3, along with their stable transfectants, were cultured at 37 °C and 5% CO2 in DMEM: F-12 (1:1) (Dulbecco's Modified Eagle Medium and Ham's F-12 Nutrient Mixture, 1:1 ratio, Invitrogen, Carlsbad, CA, USA) medium enriched with 10% fetal bovine serum (FBS) (ATLANTA, Flowery Branch, GA, USA), 2 mM L-glutamine (Corning, NY, USA), and 25 mg/mL gentamycin (Corning, NY, USA). For 5637 cells, monolayer cultivation was achieved using DMEM:F-12 (1:1) with 10% heat-inactivated FBS, under the same conditions, adhering to protocols established in our earlier research [35]. DNA authentication of all cell lines, both pre and post-research use, was performed at Genetica DNA Laboratories (Burlington, NC, USA) utilizing the PowerPlex 16 HS System, in accordance with established protocols from earlier research [8, 10]. Transfection procedures utilized PolyJet DNA In Vitro Transfection Reagent (SignaGen Laboratories, Rockville, MD, USA), introducing 1 μg of plasmid per well into 6-well plates, adhering to the supplied guidelines. Cells that survived antibiotic selection were subsequently grouped to form stable transfectant pools, consistent with methodologies outlined in prior studies [8, 10].

## Reagents, plasmids, and antibodies

The reagents used, including the dual luciferase assay kit and TRIzol, along with the SuperScript First Strand Synthesis system, were procured from Promega (Madison, WI, USA) and Invitrogen (Grand Island, NY, USA), respectively. The substrate for the luciferase assay was also obtained from Promega (Madison, WI, USA). ISO, exceeding 99% purity, was acquired from Rochen Pharma (Shanghai, China), and prepared in DMSO to a stock concentration of 20 mM. The SNHG1 promoter region was successfully amplified, cloned, and integrated into the pGL3.0 Basic vector (Ambion, Austin, TX, USA) using KpnI and HindIII restriction sites provided by New England Biolabs (Beverly, MA, USA). A point mutation was introduced at the SOX2 binding site of the SNHG1 promoter in the luciferase reporter using the following sequence-specific primers: Sense: 5'-GTT CAG GTG GCG CTT ACA CTA CGC CCT TCC-3' antisense: 5'-GGA AGG GCG TAG TGT AAG CGC CAC CTG AAC-3'. The human *SOX2* promoter region was amplified and engineered into the pGL3.0 Basic vector (Ambion, Austin, TX, USA) at the KpnI and HindIII sites (New England Biolabs, Beverly, MA, USA), utilizing specific primers as following: forward, 5'-CGG GGT ACC GAG GCT TTG TTT GAC TCC GTG T-3'; reverse, 5'-CCC AAG CTT GAG GCA AAC TGG AAT CAG GAT C-3'. The characterization of the human *SOX2* mRNA 3'-UTR luciferase construct has been detailed in preceding publications [30, 31]. A point mutation was introduced into the miR-129 binding site within the 3'-UTR of SOX2 mRNA in the luciferase reporter, employing the following specific primers: sense: 5'-ACT GTT AAA AGC TTT AAT GGC CAT GCA GGT-3'; antisense: 5'-ACC TGC ATG GCC ATT AAA GCT TTT AAC AGT-3'. Human SNHG1 full sequences were cloned downstream of the ZsGreen (Zoanthus sp. green fluorescent protein) gene in the pmR-ZsGreen1 vector (Takara Bio, Otsu, Shiga, Japan). The SOX2 overexpression construct pSin-EF2-SOX2 and the Myc-DNMT3b overexpression construct were purchased from Addgene (Cambridge, MA, USA). Utilizing PCR, the miR-129-5p overexpression construct was generated with a 250 bp segment harboring the miR-129-5p precursor, and subsequently integrated into the pcDNA3.1 vector at BamHI and EcoRI sites (New England Biolabs, Beverly, MA, USA). The miR-129 inhibitor and short hairpin RNA constructs for MMP-2, MMP-9, and PTEN, along with their respective scrambled controls, were acquired from Abm (Richmond, Canada) and Open Biosystems (Pittsburgh, PA, USA), respectively. Antibodies targeting MMP-2(Cat#sc-13594), E2F1(Cat#sc-251), HSF1(Cat#sc-17757), SOX2(Cat#sc-365823), Elk-1(Cat#sc-365876), Sp1(Cat#sc-420), DNMT3a(Cat#sc-373905), and GAPDH(Cat#sc-32233) were sourced from Santa Cruz Biotechnology (Santa Cruz, CA, USA); antibodies against MMP-9(Cat#10,375–2-AP) were purchased from Proteintech Group (Chicago, IL, USA); additionally, antibodies for DNMT3b(Cat#GTX129127), DNMT1(Cat#GTX116011), TET1(Cat#GTX124207), and TET2(Cat#GTX124205) were acquired from Gene Tex (Irvine, CA, USA); and specific antibodies targeting p-STAT3 Ser727(Cat#9134) and PTEN(Cat#9188) were obtained from Cell Signaling Technology (Beverly, MA, USA).

## RT-PCR and real-time qPCR

Total RNA extraction was performed using TRIzol reagent (Invitrogen, USA), followed by isopropyl alcohol precipitation and 75% ethanol purification, as per the manufacturer's protocol. For cDNA synthesis, 5 mg of RNA was processed with oligo(dT) primers utilizing the SuperScript First-Strand Synthesis System (Invitrogen). Additionally, for details of the primers used in RT-qPCR, please refer to Supplementary Materials. Following the Fast SYBR Green Master Mix kit's protocol (Applied Biosystems, Foster City, CA, USA), real-time PCR was performed on a 7900HT Fast Real-Time PCR System (Applied Biosystems) using previously synthesized cDNA, consistent with methodologies outlined in prior studies [32]. Total microRNA extraction was conducted using the miRNeasy Mini Kit (Qiagen, Valencia, CA), with subsequent reverse transcription facilitated by the miScript II RT Kit (Qiagen, Valencia, CA), and qPCR executed using the miScript PCR Starter Kit (QIAGEN), adhering to the prescribed protocols. Primers for specific miRNAs were obtained from Invitrogen (Grand Island, NY, USA), employing U6 as the normalization control. Cycle threshold (Ct) values were ascertained post-analysis, with relative miRNA expression quantified via the 2^−ΔΔCT^ method, aligning with previously established protocols [8].

## Western blot analyses

5637 and UM-UC-3 cell lines, along with their respective transfectants, were plated in six-well plates and cultured in standard medium until achieving 70%-80% confluence. Whole-cell extracts were then prepared with cell lysis buffer comprising 10 mM Tris–HCl [pH 7.4] (Sigma‒Aldrich, Steinheim, Germany), 1% SDS (Sigma‒Aldrich, Steinheim, Germany), and 1 mM Na3VO4 (Fisher Scientific Co., Rochester, NY, USA). Protein quantification was conducted using the NanoDrop (2000) spectrophotometer (Thermo Scientific, Waltham, MA, USA), followed by separation via SDS-PAGE and transfer onto polyvinylidene fluoride membranes (Bio-Rad, Hercules, CA, USA), ensuring precise protein analysis. Detection of target protein bands, following incubation with primary antibodies, was achieved using the Typhoon FLA 7000 (GE Healthcare, Chicago, IL, USA) with an alkaline phosphatase-linked secondary antibody coupled with an enhanced chemifluorescence detection system. Consistency of Western blotting results was ensured by replicating experiments a minimum of three times. The figures display representative blots, with densitometry performed via ImageJ (NIH, Bethesda, MD, USA) to quantitatively assess protein levels relative to loading controls. Data are represented as mean values ± standard deviations from samples in triplicate.

## Cell migration and invasion assays

In vitro migration and invasion assays were performed using Transwell chambers and Matrigel-coated chambers, respectively, following the protocols provided by manufacturer (BD Biosciences, Bedford, MA), consistent with previously described methods [12, 33]. Briefly, 3 × 10^4^ cells were seeded onto insert filters in triplicate, using 400 μL of serum-free DMEM or DMEM: F-12 (1:1). These inserts were then placed in wells containing 1 mL of medium supplemented with 10% FBS and incubated for 24 h. Post-incubation, non-migratory cells on the upper surface were removed with a cotton swab. The filters were subsequently fixed in methanol and stained with Giemsa for visualization. Migrated cells on the underside of the insert were counted using an Olympus DP71 microscope (Olympus, Center Valley, PA) across eight randomly selected fields at 50 × magnification. Quantification of migrated and invasive cells was achieved using ImageJ software (NIH, Bethesda, MD, USA), with results being representative of data collected from three independent experiments.

## Anchorage-independent Growth Assay

The ability for anchorage-independent growth was assessed using a soft agar assay, following methodologies detailed in our prior publications [8, 10]. For the assay, cells were prepared by mixing with ISO to a final concentration of 20 μmol/L, or a 0.1% DMSO vehicle control, and suspended in 2% FBS Basal Medium Eagle (BME) containing 0.33% agar. This suspension was then seeded over a basal layer consisting of 0.5% agar and 2% FBS/BME in six-well plates. Following a 3-week incubation at 37 °C in a 5% CO2 atmosphere, colonies comprising more than 32 cells were counted. Colony formation is expressed as the number of colonies per 10^4^ cells, with data presented as mean ± SD from three independent experiments.

## DNA Extraction, Bisulfite DNA Modification, and Methylation-Specific PCR

Genomic DNA was isolated from 5637 cells using the DNeasy Blood & Tissue Kit (Qiagen, Gaithersburg, MD, USA), following the kit's specific protocols. Subsequent sodium bisulfite modification and purification of the DNA were conducted using the EpiTect Bisulfite Kit (Qiagen, Gaithersburg, MD, USA), which specifically targets unmethylated cytosines for conversion. Following sodium bisulfite treatment, the genomic DNA underwent methylation-specific PCR (MSP), employing an optimized protocol detailed in previous studies [34]. Furthermore, the primers used for the miR-129 MSP are detailed in Supplementary Materials.

## Luciferase Assay

Cells were transfected with the specified luciferase reporter alongside the pRL-TK vector (Promega, Fitchburg, WI, USA) serving as an internal control. Luciferase activity was quantified using a microplate luminometer, following protocols established in prior research [8].

## Statistical analysis

The student’s t-test assessed significance between control and treatment groups, with data shown as mean ± standard deviation across a minimum of three distinct experiments. A *p*-value < 0.05 signified statistical significance among the groups compared.

## Results

### SNHG1 downregulation was crucial for the *ISO*-mediated inhibition of invasion and growth in human BC cells

**Fig. 1:**
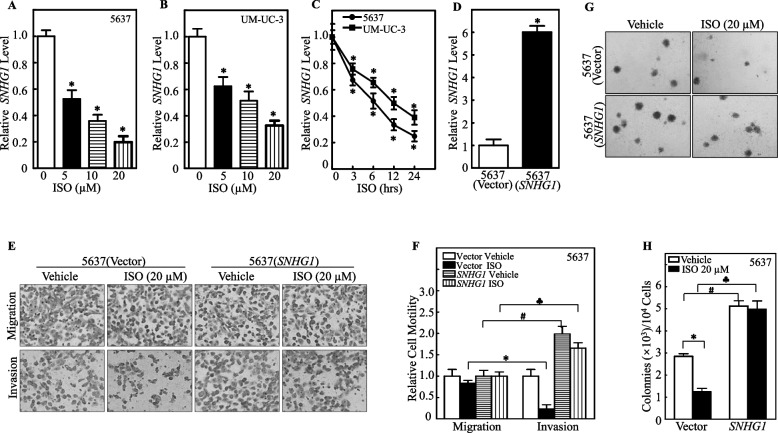
ISO inhibited invasion and anchorage-independent growth of human BMIBC cells via SNHG1 downregulation. **A**-**B **SNHG1 expression levels were evaluated by RT-qPCR in 5637 (**A**) and UM-UC-3 (**B**) cells treated with ISO at indicated doses for 24 h. **C** SNHG1 levels in 5637 and UM-UC-3 cells treated with 20 μM ISO at indicated time points. **D** SNHG1 expression in 5637 (vector) vs. 5637 (SNHG1) cells. **E**–**F** Migration and invasion assays were conducted on 5637 (SNHG1) and 5637 (vector) cells treated with 0.1% DMSO or 20 μM ISO for 24 h. Representative images are shown in (**E**), and quantified invasion normalized to the migration control is presented in (**F**). **G**-**H** Colony formation in soft agar in 5637 (SNHG1) and 5637 (Vector) cells with ISO at various concentrations; representative images (**G**) and colonies counted per 10^4^ cells (H). Data are mean ± SD from three experiments. *, #, and ♣ indicate significant differences among corresponding groups (*p* < 0.05)

Numerous studies have demonstrated that SNHG1 is upregulated in various types of cancers. In addition, aberrant expression of SNHG1 contributes to the proliferation, metastasis, migration and invasion of cancer cells [16]. 5637 and UM-UC-3 are two well-known basal muscle-invasive bladder cancer (BMIBC) cell lines that have been widely used to study BMIBC cell characteristics and the therapeutic activity of many anticancer drugs [30–33, 35]. To test the potential effect of ISO on SNHG1 expression, both 5637 and UM-UC-3 cells were treated with ISO at various concentrations (0, 5, 10, and 20 µM) for 24 h or with 20 μM ISO for different time periods as indicated, and the levels of SNHG1 were evaluated by real-time PCR. As shown in Fig. 1A-1C, SNHG1 expression was dramatically downregulated in a dose- and time-dependent manner in both cell lines. Compared with UM-UC-3 cells, 5637 cells appeared to be slightly more sensitive to ISO-induced SNHG1 inhibition and were used to further study the functional relevance and mechanism of SNHG1 downregulation. Next, we stably transfected the SNHG1 expression construct into 5637 cells and analyzed the changes in BMIBC cell migration, invasion and growth. Ectopic expression of SNHG1 was confirmed by real-time PCR (Fig. 1D). ISO treatment dramatically attenuated invasion and anchorage-independent growth without affecting migration in 5637 cells. However, ectopic expression of SNHG1 in 5637 cells specifically reversed the ISO-mediated inhibition of BMIBC invasion and cell anchorage-independent growth (Fig. 1E-1H). Taken together, these data indicate that ISO is a novel natural compound that specifically inhibits human BMIBC cell invasion and anchorage-independent growth by inhibiting SNHG1 expression.

### MMP-2/MMP-9 suppression and PTEN induction functioned as downstream effectors of SNHG1 for the *ISO*-mediated inhibition of BMIBC invasion and growth

**Fig. 2:**
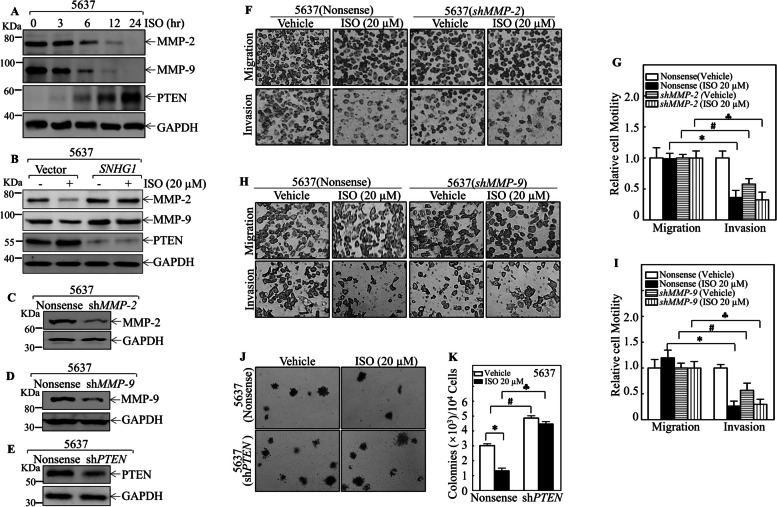
SNHG1 downregulation contributed to ISO-mediated suppression of MMP-2/MMP-9 and induction of PTEN in BMIBC cells. **A** 5637 cells treated with 20 μM ISO at the indicated time points were analyzed by western blotting for MMP-2, MMP-9, and PTEN; GAPDH served as control. **B** Western blot of proteins from 5637 (SNHG1) and 5637 (Vector) cells treated with 0.1% DMSO or 20 μM ISO for 24 h, with GAPDH as control. (**C**-**E**) Protein expression in indicated stable transfectants was analyzed by Western blot, normalized to GAPDH. **F**-**I** The invasion and migration abilities of 5637 (nonsense) vs. 5637 (shMMP-2) and 5637 (nonsense) vs. 5637 (shMMP-9) cells, with or without ISO treatment, were assessed (**F** & **H**). Invasion ability was quantified and presented in (**G** & **I**). **J**-**K** Colony formation in 5637 (shPTEN) vs. 5637 (nonsense) cells in soft agar with or without ISO treatment was quantified. Data are mean ± SD from three experiments. *, # and ♣ indicate significant differences (*p* < 0.05)

To investigate the SNHG1 downstream effectors responsible for ISO suppression of human BMIBC cell invasion and cell anchorage-independent growth, we first analyzed the expression of MMP-2 and MMP-9, which are positive regulators of cancer invasion, and of PTEN, a tumor suppressor that negatively regulates cancer cell proliferation and growth [36], in 5637 cells after ISO treatment using Western blotting. The results indicated that MMP-2 and MMP-9 were dramatically decreased in 5637 cells after ISO treatment, while PTEN was markedly increased in 5637 cells following ISO treatment (Fig. 2A). However, ectopically expressing SNHG1 in 5637 cells not only reversed the ISO-mediated inhibition of MMP-2 and MMP-9 expression but also abolished the ISO-induced increase in PTEN expression in 5637 cells (Fig. 2B), suggesting that MMP-2, MMP-9 and PTEN are downstream targets of SNHG1. To evaluate the functional role of MMP-2, MMP-9 and PTEN in the ISO-mediated inhibition of BMIBC cell invasion and anchorage-independent growth, 5637 cells were transfected with human MMP-2, MMP-9, and PTEN constructs, and these proteins were successfully knocked down, as determined by Western blotting (Fig. 2C-2E). Interestingly, knockdown of MMP-2 or MMP-9 in 5637 cells resulted in significantly reduced invasion, similar to that observed following ISO treatment (Fig. 2F-2I), whereas shPTEN reversed the ISO-mediated inhibition of BMIBC anchorage-independent growth in 5637 cells (Fig. 2J-K). These results indicate that SNHG1 reverses ISO-mediated inhibition of BMIBC cell invasion and anchorage-independent growth by modulating the levels of MMP-2, MMP-9 and PTEN.

### SOX2 reduction was responsible for the *ISO*-induced downregulation of SNHG1 transcription

**Fig. 3:**
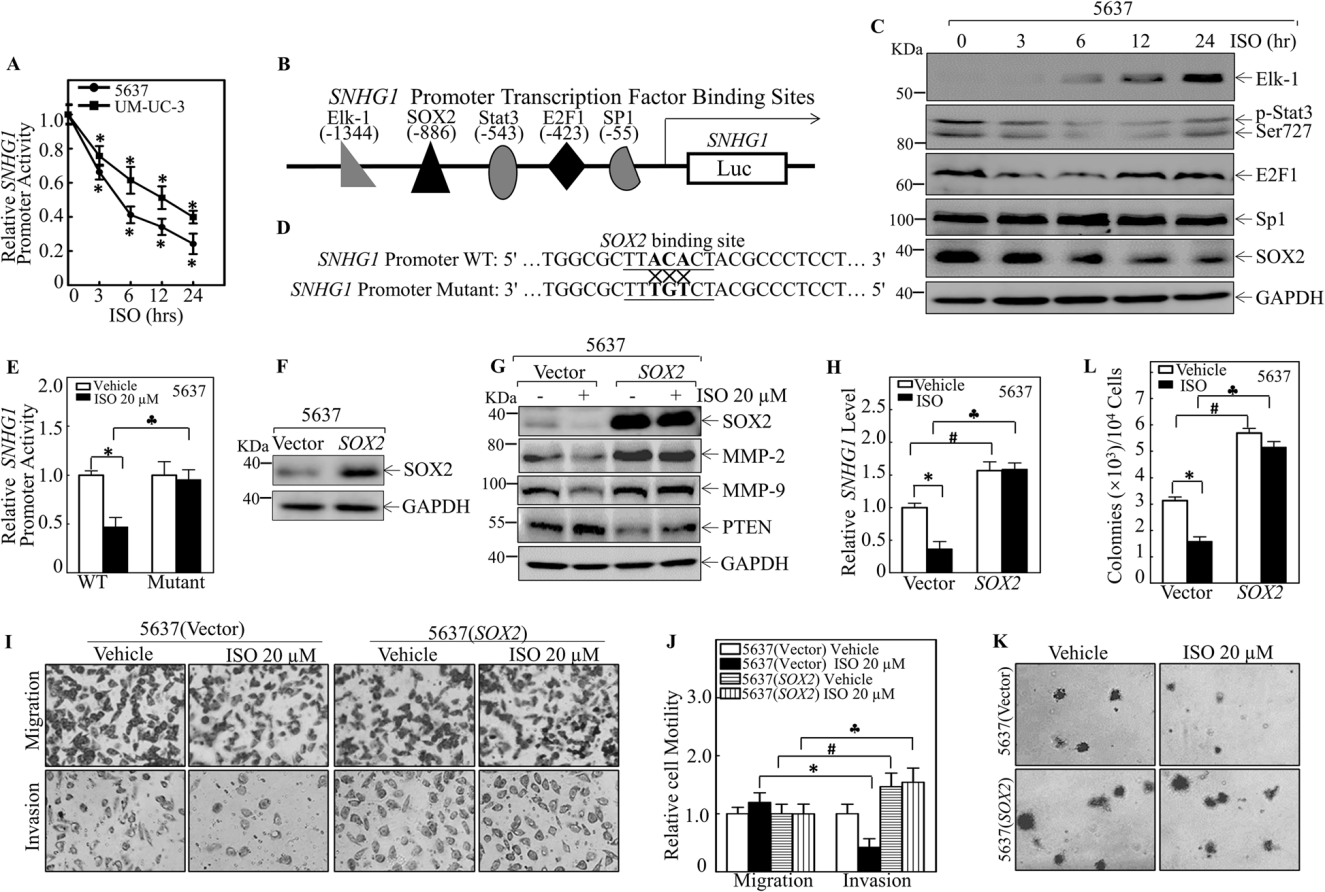
SOX2 suppression mediated ISO-induced SNHG1 downregulation in BMIBC cells. (A) Wild-type SNHG1 promoter-driven luciferase reporter was cotransfected with pRL-TK into 5637 or UM-UC-3 cells; luciferase activity was measured 24 h post-transfection. TK served as an internal control. *Significantly different from the control group, *p* < 0.05. (B) Predicted transcription factor-binding sites in the SNHG1 promoter. (C) Transcription factor expression in 5637 cells treated with ISO at indicated times. (D) Schematic of SOX2 binding site in wild-type and mutant SNHG1 promoters. (E) 5637 cells cotransfected with wild-type or mutant SNHG1 promoter reporters and pRL-TK. (F) Western blot analysis of SOX2 expression in 5637 (SOX2) and 5637 (Vector) cells; GAPDH as control. (G) Western blot analysis of indicated proteins in cells with/without ISO treatment; GAPDH for normalization. (H) Relative SNHG1 levels in 5637 (SOX2) and 5637 (Vector) cells with 20 µM ISO or 0.1% DMSO were assessed by RT-qPCR. (I & J) Invasion abilities of 5637 (Vector) and 5637 (SOX2) cells with 20 µM ISO or 0.1% DMSO (I); relative invasion plotted (J). *p < 0.05, #*p* < 0.05, ♣*p* < 0.05 vs. respective controls. (K & L) Colonies of 5637 (SOX2) and 5637 (Vector) cells in soft agar with 20 µM ISO or 0.1% DMSO (K). Colony counts shown in (L). *p < 0.05, #*p* < 0.05, ♣*p* < 0.05 vs. respective controls

Treatment of 5637 and UM-UC-3 cells with ISO resulted in a significant reduction in SNHG1 RNA levels in a time-dependent manner (Fig. 1C), and stable ectopic expression of SNHG1 reversed the ISO-mediated inhibition of invasion and anchorage-independent growth in BMIBC cells (Fig. 1G-1H), suggesting that ISO might inhibit SNHG1 RNA expression at the transcriptional level. To test this hypothesis, a SNHG1 promoter luciferase reporter was transfected into 5637  and UM-UC-3 cells, and the resulting promoter transcriptional activity was analyzed in the absence or presence of ISO. Significant inhibition of SNHG1 promoter activity following ISO treatment was observed in both 5637 and UM-UC-3 cells (Fig. 3A), suggesting that ISO downregulated SNHG1 expression at the transcriptional level in both 5637 and UM-UC-3 cells.

To further investigate the potential upstream regulators mediating ISO-mediated inhibition of SNHG1 transcription, bioinformatics analysis of the SNHG1 promoter was performed, and multiple binding sites for various transcription factors were identified, as shown in Fig. 3B. Among these transcription factors, SOX2 exhibited a time-dependent reduction in 5637 cells following ISO treatment, whereas other transcription factors, including Elk1, p-Stat3 Ser727, E2F1, and Sp1, did not show consistent alterations as SNHG1 (Fig. 3C). To test whether SOX2 is a key ISO downstream transcription factor responsible for SNHG1 transcriptional downregulation, a point mutation was introduced into the SOX2 binding site of the SNHG1 promoter (Fig. 3D). When transfected into 5637 cells, the mutation in the SOX2 binding site completely abolished the ISO-mediated inhibition of SNHG1 promoter transcriptional activity (Fig. 3E). These results indicate that the SOX2 binding site in the SNHG1 promoter is crucial for ISO-mediated inhibition of SNHG1 promoter transcriptional activity, further demonstrating that SOX2 downregulation is responsible for SNHG1 inhibition by ISO.

To investigate the functional relevance of SOX2 downregulation in the context of ISO inhibition, we generated a 5637 cell line with stable overexpression of SOX2 and quantified the protein levels using Western blot analysis (Fig. 3F). ISO treatment did not have a marked inhibitory effect on exogenously expressed SOX2 protein expression, whereas it dramatically inhibited endogenous SOX2 protein expression in 5637 cells (Fig. 3G). Consistently, in comparison to 5637(Vector) cells, SOX2 overexpression abolished SNHG1 inhibition by ISO treatment (Fig. 3H). Moreover, compared with ISO treatment, ectopic expression of SOX2 restored MMP-2 and MMP-9 expression and abolished PTEN protein expression (Fig. 3G). Furthermore, SOX2 overexpression reversed ISO-mediated inhibition of BMIBC cell invasion (Fig. 3I & 3J) and anchorage-independent growth (Fig. 3K & 3L) in 5637 cells. These results demonstrate that SOX2 downregulation contributes to the ISO-mediated inhibition of BMIBC invasion and anchorage-independent growth in 5637 cells.

### miR-129 repressed SOX2 by directly interacting with its mRNA 3'-UTR

**Fig. 4:**
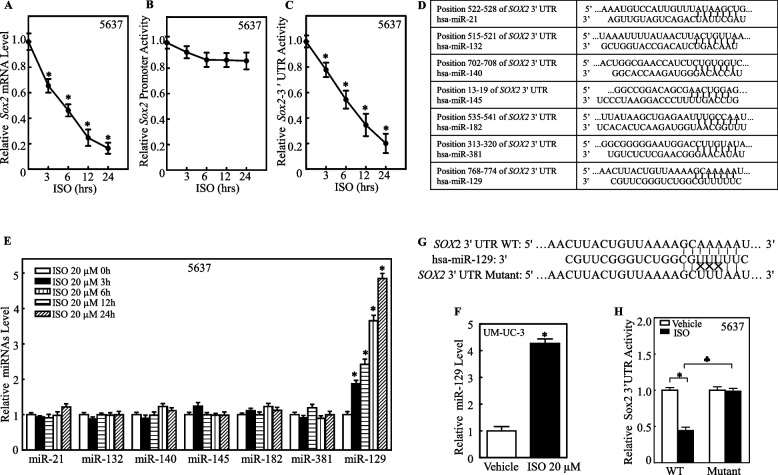
miR-129 induced by ISO destabilizes SOX2 mRNA by interacting with its 3′UTR. **A** Relative SOX2 mRNA levels in 5637 cells treated with 20 μM ISO at indicated times by RT-qPCR. (**B**) Wild-type SOX2 promoter-driven luciferase reporter cotransfected with pRL-TK into 5637 cells. (**C**) 5637 cells were cotransfected with wild-type SOX2 mRNA 3′-UTR luciferase reporters and pRL-TK, followed by treatment with 20 μM ISO. TK served as the internal control. Luciferase activity is presented relative to SOX2 promoter activity. *p < 0.05 vs. 0.1% DMSO group. Bars represent mean ± SD from three independent trials. (**D**) Potential miRNA binding sites in SOX2 mRNA 3′-UTR from TargetScan database. (**E**) Relative miRNA levels in 5637 cells treated with 20 μM ISO at indicated times by RT-qPCR. (**F**) Relative miR-129 levels in UM-UC-3 cells with/without 20 μM ISO treatment. (**G**) miR-129 binding site in SOX2 mRNA 3′-UTR and mutants. (**H**) 5637 cells were cotransfected with wild-type or mutant SOX2 mRNA 3′-UTR luciferase reporters and pRL-TK, followed by treatment with 0.1% DMSO or 20 μM ISO for 24 h. Relative SOX2 mRNA 3′-UTR activity is presented. Data are shown as mean ± SD from three independent tests. *p < 0.05, **♣***p* < 0.05 among corresponding groups

To understand why SOX2 was downregulated, we first assessed the SOX2 mRNA level in 5637 cells treated with 20 µM ISO at various time points. The results indicated that SOX2 mRNA expression was significantly inhibited in a time-dependent manner after ISO treatment (Fig. 4A), suggesting that ISO might alter SOX2 mRNA expression at the transcriptional level and/or mRNA stability. To test whether ISO inhibits SOX2 expression at the transcriptional level, a luciferase reporter containing the *SOX2* promoter was transfected into 5637 cells, and the resulting promoter activity was analyzed in the presence or absence of ISO treatment. ISO did not significantly affect SOX2 promoter activity (Fig. 4B), suggesting that ISO is unlikely to inhibit SOX2 at the transcriptional level. Therefore, a *SOX2* 3'-UTR luciferase reporter was transfected into 5637 cells to test whether ISO affects SOX2 mRNA 3'-UTR activity. As shown in Fig. 4C, ISO treatment significantly inhibited SOX2 3'-UTR activity in 5637 cells, suggesting that miRNAs might reduce SOX2 mRNA stability upon ISO treatment. The potential miRNA binding sites in the *SOX2* 3'-UTR luciferase reporter were analyzed using “targetscan.org”, which revealed the binding sites for seven miRNAs, namely, miR-21, miR-132, miR-140, miR-145, miR-182, miR-129 and miR-381 (Fig. 4D). The relative expression levels of these miRNAs were evaluated in ISO-treated 5637 cells, which revealed that only the abundance of miR-129 was significantly increased in 5637 cells after ISO treatment (Fig. 4E). A similar increase in the miR-129 level was detected in ISO-treated UM-UC-3 cells (Fig. 4F), suggesting that SOX2 might be a direct target of miR-129. To test the effect of miR-129 on SOX2 expression, a SOX2 3'-UTR luciferase reporter containing a point mutation in the miR-129 binding site, as indicated in Fig. 4G, was transfected into 5637 cells. As shown in Fig. 4H, a point mutation in the miR-129 binding site completely abolished the ISO-mediated reduction in 3'-UTR luciferase activity in 5637 cells, suggesting that miR-129 mediates the ISO-mediated inhibition of SOX2 mRNA 3'-UTR activity and further SOX2 mRNA expression.

**Fig. 5:**
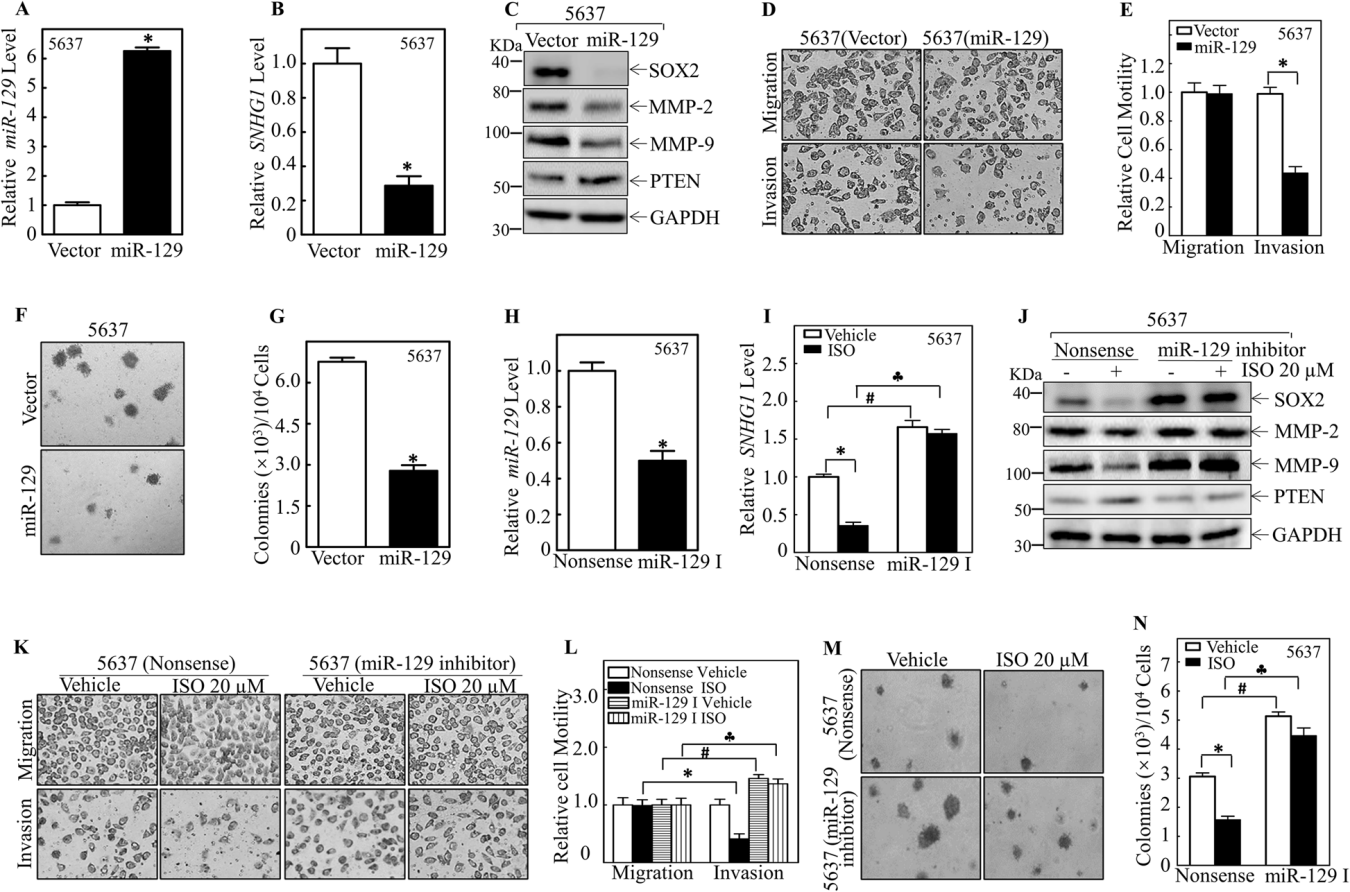
miR-129 suppresses invasion and anchorage-independent growth in 5637 cells. **A**-**B** miR-129 (**A**) and SHNG1 (**B**) expression in 5637 (vector) and 5637 (miR-129) cells by RT-qPCR. **C** Protein expression in 5637 (vector) and 5637 (miR-129) cells; GAPDH for normalization. (**D**-**E**) The invasion abilities of 5637 (vector) and 5637 (miR-129) cells were assessed using the Transwell assay (**D**), with relative invasion plotted in (**E**). (**F**-**G**) Colony images in soft agar (**F**), and colony count per 10^4^ cells (**G**). (**H**-**I**) miR-129 (**H**) and SHNG1 (**I**) expression in 5637 (miR-129 inhibitor) and 5637 (nonsense) cells by RT-qPCR. (**J**) Protein expression in 5637 (nonsense) and 5637 (miR-129 inhibitor) cells with/without ISO treatment; GAPDH for normalization. (**K**-**L**) The invasion abilities of 5637 (vector) and 5637 (miR-129 Inhibitor) cells, with or without ISO treatment, were assessed using the Transwell assay (**K**), and the relative invasion ability was plotted in (**L**). (**M**–**N**) Colony images in soft agar with/without ISO (M), and colony count per 10^4^ cells (**N**). Values are mean ± SD from three trials. *, #, and **♣** indicate significant differences (*p* < 0.05) among treatment groups

To further confirm the important role of miR-129 in ISO-induced SNHG1 inhibition as well as its anticancer activity, we established a 5637 cell line with stable expression of miR-129, and the relative expression level of miR-129 was determined by real-time PCR (Fig. 5A). As shown in Fig. 5B-5C, similar to ISO treatment, miR-129 overexpression not only inhibited SNHG1 expression but also consistently altered SOX2, MMP-2, MMP-9, and PTEN protein levels in 5637 cells. Further study demonstrated that ectopic expression of miR-129 attenuated BMIBC cell invasion and anchorage-independent growth in 5637 cells (Fig. 5D-G). In contrast, knockdown of miR-129 using its specific inhibitor (Fig. 5H) abolished the ISO-induced downregulation of SNHG1 (Fig. 5I), SOX2, MMP-2, and MMP-9 and the ISO-induced upregulation of PTEN expression in 5637 cells (Fig. 5J). Moreover, the introduction of a miR-129 inhibitor into 5637 cells also specifically reversed the ISO-mediated inhibition of BMIBC invasion and cell anchorage-independent growth (Fig. 5K-N). These results demonstrate that miR-129 induction by ISO is responsible for the inhibition of SNHG1, SOX2, MMP-2, and MMP-9 expression and the induction of PTEN expression in 5637 cells, further mediating the inhibition of BMIBC invasion and anchorage-independent growth in 5637 cells.

### DNMT3b is an upstream regulator of the suppressive effect of miR-129 on SNHG1 expression

**Fig. 6:**
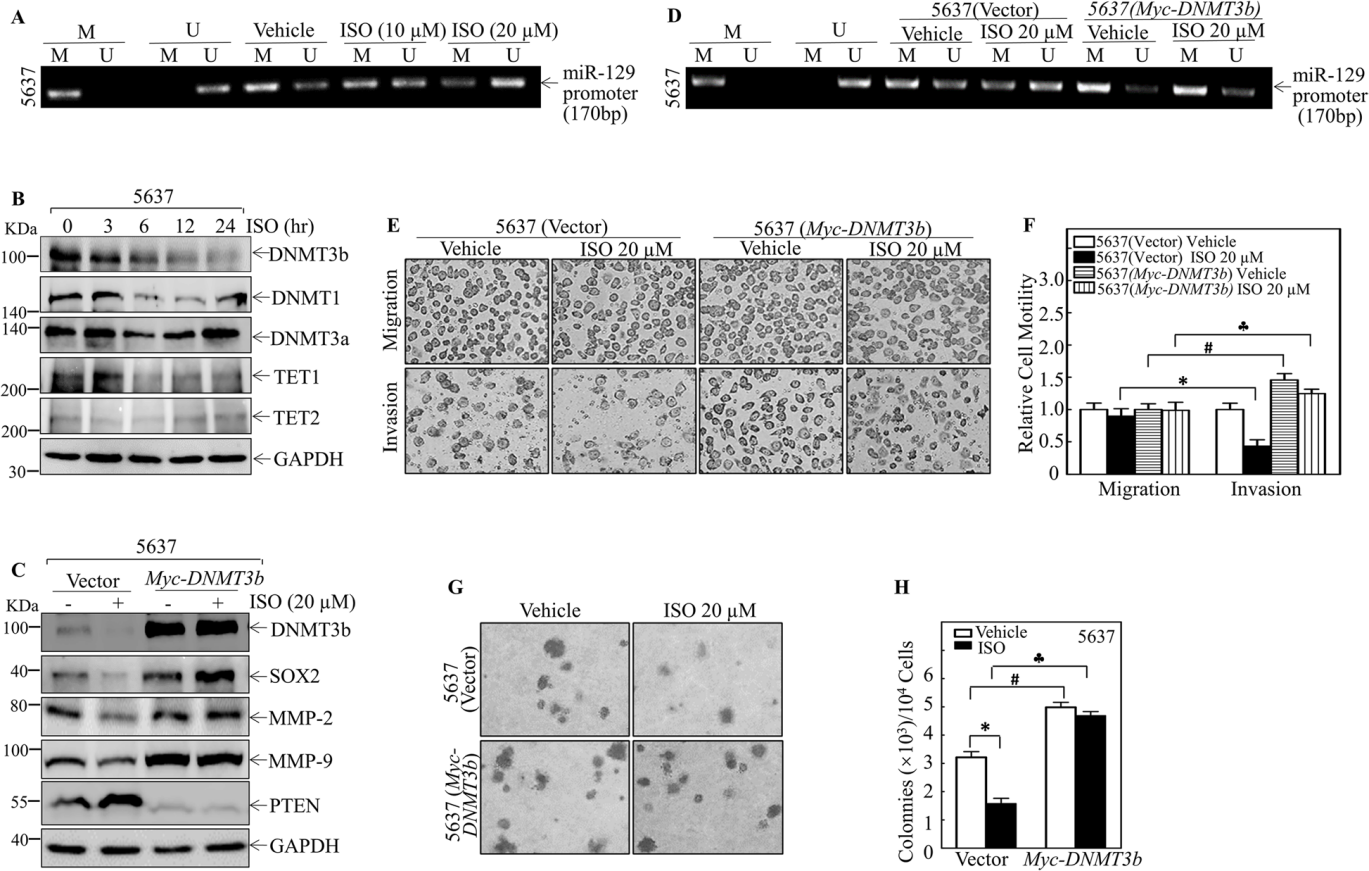
ISO-induced DNMT3b downregulation led to miR-129 promoter hypomethylation, suppressing BMIBC invasion and growth. **A** Methylation status of the miR-129 promoter in 5637 cells treated with ISO at indicated concentrations was determined by MS-PCR. **B** Expression of indicated proteins in 5637 cells treated with ISO at various time points; GAPDH served as a normalization reference. TET1 and TET2 detection involved membrane trimming prior to antibody incubation. **C** Protein expression in 5637 (vector) and 5637 (Myc-DNMT3b) cells with or without ISO treatment; GAPDH was used for normalization. DNMT3b blots were rescanned from X-ray films for enhanced quality. **D** Methylation status of the miR-129 promoter in 5637 (vector) and 5637 (Myc-DNMT3b) cells with/without ISO was determined by MSP. (**E**–**F)** Invasion abilities of 5637 (vector) and 5637 (Myc-DNMT3b) cells with/without ISO evaluated by Transwell assay (**E**), with relative invasion plotted (**F**). **G**–**H** Colony images in soft agar with/without ISO treatment (**G**), and colony count per 10^4^ cells (**H**). Data are mean ± SD from triplicate samples. *, #, and **♣** indicate significant differences (*p* < 0.05) among treatment groups

**Fig. 7:**
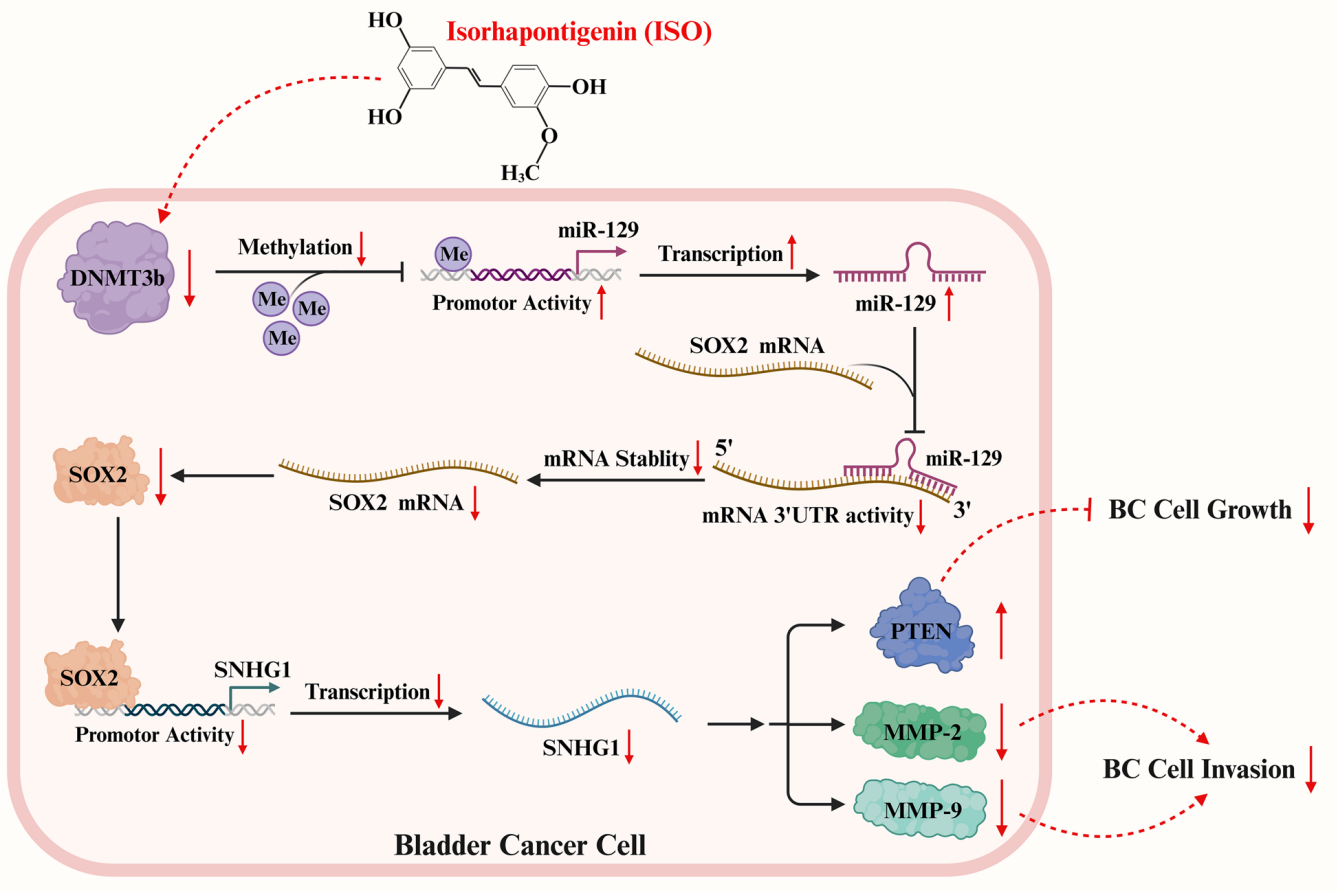
Mechanistic insights into ISO-mediated suppression of BMIBC cell invasion and growth. ISO treatment suppresses SNHG1 transcription through the DNMT3b/miR-129/SOX2 axis, which leads to a reduction in MMP-2 and MMP-9 protein levels and an increase in PTEN protein expression, ultimately attenuating invasion and anchorage-independent growth of BIMBC cells

It has been reported that miR-129 functions as a tumor suppressor in cancer cells, and miR-129 promoter hypermethylation is an important mechanism associated with the loss of miR-129 expression in tumors [37, 38]. To test whether ISO alters the methylation status of the miR-129 promoter, the methylation levels of a CpG-rich region (~ 170 bp) upstream of the miR-129 transcription initiation site were analyzed by MSP in 5637 cells treated with various doses of ISO for 24 h. As shown in Fig. 6A, ISO treatment resulted in a dose-dependent decrease in methylated DNA (M), which was accompanied by the upregulation of unmethylated DNA (U) in 5637 cells. DNA methylation is regulated by a balance between DNA methyltransferases (DNMTs) and demethylases, such as the TET family proteins [39–41]. To explore the possible enzymes that mediate ISO-induced hypomethylation of the miR-129 promoter, the levels of three DNMTs and two TETs were analyzed in 5637 cells treated with ISO at different time points. As shown in Fig. 6B, ISO treatment for 24 h specifically inhibited the protein expression of DNMT3b but not that of DNMT1 or DNMT3a. To determine whether DNMT3b regulates miR-129, DNMT3b was stably transfected into 5637 cells, and miR-129 promoter methylation and SOX2, MMP-2, MMP-9 and PTEN expression were analyzed in the presence or absence of ISO. As shown in Fig. 6C, ectopic expression of DNMT3b abolished ISO-induced SOX2 downregulation, MMP-2 and MMP-9 inhibition and PTEN upregulation. Consistently, overexpressing DNMT3b rescued the miR-129 promoter hypomethylation induced by ISO treatment (Fig. 6D). Further study demonstrated that ectopic expression of DNMT3b reversed the ISO-mediated inhibition of BMIBC cell invasion and anchorage-independent growth of 5637 cells (Fig. 6E-H). Taken together, these results strongly indicated that DNMT3b downregulation contributes to ISO-induced miR-129 promoter hypomethylation, which leads to the induction of miR-129 expression, consequently resulting in reduced SOX2, SNHG1, MMP-2 and MMP-9 expression as well as increased PTEN protein expression, ultimately attenuating BMIBC cell invasion and anchorage-independent growth, as summarized in Fig. 7.

## Discussion

MIBC poses a significant threat to human health due to its aggressive nature and poor prognosis, often resulting in a high mortality rate, which presents a substantial therapeutic challenge [4]. Therefore, elucidating the mechanisms underlying MIBC cell invasion and cell growth capacities as well as developing new anticancer compounds is urgently needed. Previous studies have shown that ISO elicits multiple anticancer activities, including the inhibition of cell invasion [9, 12, 13] and proliferation [31, 42], as well as the promotion of apoptosis [6] and autophagy [10]. Despite its promising profile, the specific anticancer effects of ISO on MIBC and the underlying mechanisms remain largely unexplored. Consequently, our current study demonstrates that ISO inhibits the growth and invasion of MIBC cells by downregulating SNHG1 through the DNMT3b/miR-129/SOX2 pathway. These findings suggest that ISO is a promising therapeutic compound for MIBC treatment, corroborated by previous reports on its anticancer effects both in vivo and in vitro [8–13].

In this study, we discovered for the first time that ISO treatment downregulates DNMT3b protein levels, reduces methylation of the miR-129 promoter, and increases miR-129 transcription in BMIBC cells. Emerging evidence indicates that epigenetic changes, such as DNA methylation, play a vital role in cancer development, with aberrant methylation of promoter regions leading to the dysregulation of miRNA expression in several malignant tumors [43–45]. An increasing number of studies have shown that miR-129 is a promising candidate biomarker for diagnostic and prognostic purposes in various cancers. It has been reported that miR-129 is downregulated in breast cancer [38], colorectal cancer [46], gastric cancer [46], and pancreatic cancer [46]. Additionally, Tang et al. report that miR-129 promoter hypermethylation is responsible for the downregulation of miR-129 in breast cancer [38]. Consistently, we showed that miR-129 plays a tumor suppressive role in human MIBC cells. In addition, the interplay between non-coding RNA and DNMT3b can significantly influence tumor cell fate [47]. Our study also finds that DNMT3b plays a major role in regulating miR-129 promoter methylation upon ISO treatment. This finding elucidates the epigenetic regulation of miR-129 by promoting the interaction of DNMT3b with the miR-129 promoter. Nevertheless, the underlying mechanism by which ISO inhibits DNMT3b is currently under investigation in our group.

SOX2, a transcription factor, recognized as a stem cell marker, exhibits increased expression in BC across both mouse models and human tissues [48]. Numerous studies have reported that SOX2 plays an important role in the development of various human cancers. For example, SOX2 reportedly promotes the invasion of MIBC cells [48]. Additionally, high expression of SOX2 significantly enhances the growth and invasion of ovarian cancer, breast cancer, and hepatocellular carcinoma cells [49]. SOX2 also promotes tumorigenesis and malignant transformation in prostate cancer by regulating metabolic reprogramming and enhancing glycolytic capacity [50]. Besides, SOX2 is a lineage-specific oncogene that is widely amplified and overexpressed in various squamous cell carcinomas, including esophageal and head and neck squamous cell carcinomas [51]. The amplification and overexpression of SOX2 are recognized as hallmarks of squamous cell carcinomas originating from different tissue types [51]. Futhermore, loss of miR-638 in vitro promotes cell invasion and mesenchymal-like transition by regulating SOX2 expression in colorectal carcinoma cells [52]. Our published studies demonstrate that ChlA-F, a novel conformational derivative of the isolate Cheliensisin A (Chel A), remarkably inhibits the invasive ability of human invasive BC cells through downregulation of SOX2 protein expression, indicating that SOX2 plays an oncogenic role in human BC [30]. However, to the best of our knowledge, whether SOX2 plays a role in the ISO-mediated inhibition of BC invasion has not yet been investigated. Our studies here exhibit that ISO downregulates SOX2 expression, subsequently leading to the inhibition of BMIBC cell invasion and anchorage-independent growth. Further mechanistic studies show that ISO inhibits DNMT3b expression and increases miR-129 promoter hypomethylation, leading to increased miR-129 expression and reduced SOX2 mRNA expression.

Our current study finds that ISO treatment induces significant downregulation of SNHG1 expression, which is associated with the downregulation of SOX2. SNHG1 is a novel oncogenic lncRNA located on chromosome 11, and accumulating evidence has demonstrated that SNHG1 is upregulated in various types of cancers, often promoting tumorigenesis and tumor progression [20–28]. Our recent research also indicates that BBN treatment induces primary BMIBC formation with significantly upregulation of SNHG1 in vivo [53]. Moreover, overexpression of SNHG1 is sufficient to drive malignant transformation of normal human urothelial cells and promotes tumorigenicity of human BMIBC in nude mice [53]. These findings indicate that SNHG1 functions as a significant oncogene in BC, with its aberrant expression leading to the development and progression of BMIBC, and are in line with the present study that ISO treatment induces a decrease in SNHG1 transcription levels, leading to significantly reduced invasion and anchorage-independent growth of BMIBC cells.

Cancer metastasis includes multiple steps, such as local tumor cell invasion, entry into the vasculature, cancer cell exit from circulation, and colonization at distal sites [54]. Cancer cells can invade and exhibit anchorage-independent growth, whereas many types of normal cells can migrate without invasion or anchorage-independent growth. Thus, investigating the underlying mechanisms for the migration, invasion, and anchorage-independent growth of BC cells is very important for identifying new therapeutic targets for BC management. MMP-2 and MMP-9 are two critical enzymes that mediate extracellular matrix (ECM) degradation, which is an important process that allows cancer cell invasion and metastasis [55]. Our previous studies report that ectopic expression of MMP-2 is crucial for human BC invasion [56, 57], and ISO can significantly suppress both BMIBC cell invasion in vitro and highly BMIBC formation in BBN-induced BMIBC in vivo mouse model [8, 9]. MMP-9 plays a crucial role in maintaining the extracellular matrix homeostasis of healthy bladder tissue and remodeling the extracellular matrix under pathological conditions, with its expression and activity significantly elevated in high-grade BC [58]. PTEN, a negative regulator of the PI3K/Akt signaling pathway, functions as a tumor suppressor to negatively regulate cell proliferation and growth [36]. Xu et al. report that the ubiquitination and degradation of PTEN by RNF126 leads to its reduced expression, resulting in the hyperactivation of the PI3K/Akt pathway, which further promotes the growth, invasion, and metastasis of BC cells [59]. Herein, we discover that SNHG1 inhibition by ISO treatment mediates the suppression of BMIBC cell invasion accompanied with the marked downregulation of MMP-2 and MMP-9, while SNHG1 inhibition by ISO is also associated with the attenuation of anchorage-independent growth of BMIBC cells concurrently with the upregulation of PTEN protein expression. Our results indicate that SNHG1 specifically induces BC invasion and MMP-2 expression *by increasing* its transcription and mRNA expression [57] and that PTEN upregulation is mediated by its degradation due to USP8 reduction and is responsible for SNHG1-mediated promotion of BMIBC cell anchorage-independent growth [53]. Consequently, we hypothesize that the inhibition of SNHG1 by ISO treatment, resulting in MMP-2 and MMP-9 downregulation and PTEN upregulation, plays a pivotal role in the anticancer effects of ISO, as demonstrated in both in vitro cell culture model and in vivo BBN-induced BMIBC formation.

In summary, our results demonstrate that ISO treatment downregulates SNHG1 via the DNMT3b/miR-129/SOX2 axis, consequently resulting in MMP-2 and MMP-9 suppression and PTEN induction, ultimately leading to the inhibition of BMIBC cell invasion and growth, which further supports the potential of ISO as an effective therapeutic agent for clinical treatment of BMIBC. Given that SNHG1 is significantly upregulated in human BMIBCs, this novel DNMT3b/miR-129/SOX2/SNHG1 cascade highlights its potential utilization as an informative biomarker for the prognosis of BMIBC patients. Furthermore, the identification of SNHG1 as an oncogene that promotes BMIBC cell invasion and anchorage-independent growth will also provide significant insights for further exploration of SNHG1 as a new target for the management of BMIBC patients.

## Data Availability

All data generated or analysed during this study are included in this published article.

## Abbreviations

BC: Bladder Cancer
BMIBC: Basal Muscle-Invasive Bladder Cancer
ISO: Isorhapontigenin
DNMT3b: DNA Methyltransferase 3B
miR-129: MicroRNA-129
SOX2: SRY-Box Transcription Factor 2
SNHG1: Small Nucleolar RNA Host Gene 1
PTEN: Phosphatase and Tensin Homolog
MMP-2: Matrix Metallopeptidase 2
MMP-9: Matrix Metallopeptidase 9
